# Challenges and impacts from wait times for specialist care identified by primary care providers: Results from the MAAP study cross-sectional survey

**DOI:** 10.1177/08404704231182671

**Published:** 2023-07-06

**Authors:** Emily G. Marshall, Laura Miller, Lauren R. Moritz

**Affiliations:** 1 3688 Dalhousie University, Halifax, Nova Scotia, Canada.

## Abstract

In Canada, primary care providers are the front door to other services in the health system, such as specialist care. Compared to other countries, Canadians experience long wait times for specialist referrals and appointments leading to poorer health outcomes for patients. Although there is attention paid to the impacts of these waits on patients, little is known about how long specialist care wait times impact primary care providers. As part of a larger study surveying primary care clinics in Nova Scotia, primary care providers were invited to participate in a follow-up survey on comprehensive care and specialist wait times. We thematically analyzed responses to an open text field about specialist wait times. Respondents shared experiences with challenging specialist wait times, strategies to manage patients waiting for specialist care, and recommendations for improving access to specialist care in Nova Scotia, Canada.

## Introduction

Primary care is the foundation of strong healthcare systems and, for many in Canada, Primary Care Providers (PCPs) such as family physicians or nurse practitioners are the first point of contact with the health system.^1^ Patients requiring care outside the scope of a PCPs’ practice are referred to other medical specialists by their PCP.^2,3^ PCPs therefore act as “gatekeepers” for patient access to speciality care, opening the door for patients to access needed services, similar to processes in the United Kingdom.^4^

Long wait times between PCP referrals to appointments with a specialist remain a top barrier to healthcare access in Canada.^5,6,7^ Long wait times occur across a variety of different specialities (e.g. psychiatry, orthopaedics, gastroenterology, and rheumatology^6^). Compared to other countries, studies indicate longer and more problematic wait times for specialists in Canada.^8-10^ In 2020, Canadians experienced the longest wait times among the eleven Commonwealth countries, with 62% of patients requiring specialist care waiting one month or more for access.^11^ In comparison, 55% of patients in the United Kingdom and 31% in the United States experience wait times for specialists of one month or more.^11^ Wait times are also more problematic in Canada for adults 65 years of age or older compared to other Commonwealth countries (31% wait over six days in Canada compared to 14% in the United Kingdom and 22% in the United States).^12^ Wait times for specialist care in Canada have not improved over the past decade. In 2010 and 2016, 56% of Canadians waited 4 weeks or longer for specialist care,^10^ increasing to 62% in 2020.^11^ During the COVID-19 pandemic, many specialist appointments were postponed or delayed,^13-15^ resulting in further increased wait times for specialist appointments.

Time spent waiting for initial contact with specialist care is an important period in the continuum of care,^16^ and research supports the negative impact on patient health and quality of life.^17-19^ However, little is known about the impact of these long wait times on PCP practice. Understanding the impact of long specialist wait times on PCP practice is essential to supporting the Quintuple Aim for health services, which not only includes enhancing patient experience, but also promotion of care team well-being, population health outcomes, optimizing costs, and health equity.^20^ The purpose of this study is to explore the pervasiveness and consequences of specialist wait times for patients and PCPs in Nova Scotia, Canada, and to identify recommendations for improvements.

## Method

### Study population, setting, and design

As part of the *Models and Access Atlas of Primary Care in Nova Scotia* (MAAP-NS) study, we conducted a cross-sectional, linked survey with all PCPs in Nova Scotia, Canada, between 2015 and 2019.^21^ Five hundred and sixty-six PCPs participated in the study (approximately 60% of all PCPs in the province). All respondents agreed to be contacted for a follow-up survey of nine items relating to comprehensiveness of family practice, including wait times for specialist referrals. This survey was linkable to previous survey responses. The survey was distributed between May and September 2018. This article presents findings from the follow-up survey.

### Statistical analysis

Descriptive statistics were computed from the first MAAP-NS survey for demographics including sex, age, provider type, practice model (fee for service, alternate payment plan; solo practice or interdisciplinary model), practice location, and rurality. These demographic data were linked with each PCP participating in the follow-up comprehensiveness survey. Survey responses provided by the PCPs were grouped into themes, then assigned a code number for entry into SPSS,^22^ which was used to calculate response frequency. Open-ended survey question responses were chosen to illustrate identified themes.

## Results

### Descriptive

Of the 566 PCPs who received the follow-up survey, 98 completed the survey (17.3% response rate), with 87 responding to the open field question *“How do the wait times for specialists affect the way you practice?”* These response rates are higher than most PCP surveys.^23,24^ PCP age ranged from 32 to 72 years (*M* = 50.01, *Mode* = 60), with representation across sex, provider type, practice type, and rurality (Table 1).

**Table 1.** Family physician and nurse practitioner demographics.

Variable	n (%)
Sex	
Female	51 (58.62)
Male	35 (40.23)
Missing	1 (1.15)
Provider type	
Family physician	82 (94.25)
Nurse practitioner	4 (4.60)
Missing	1 (1.15)
Practice type	
Interdisciplinary	46 (52.87)
Non-interdisciplinary	40 (45.98)
Missing	1 (1.15)
Rurality^a^	
Urban core (Halifax)	31 (35.63)
Metro fringe and satellite urban town	24 (27.59)
Middle to small town	18 (20.69)
Village	11 (12.64)
Sparse settlement	2 (2.30)
Missing	1 (1.15)

^a^Rurality defined according to Terashima, Guernsey, and Andreou’s typology.^25^

### Frequencies

Of the 87 PCP participants, there were 156 responses to the question *“How do the wait times for specialists affect the way you practice?*” Using thematic analysis, we identified nine themes: (1) pervasiveness of problematic specialist wait times; (2) managing beyond scope while waiting for specialist care; (3) consequences for patients due to specialist wait times; (4) managing patient expectations while waiting for specialist care; (5) scheduling repeat visits to meet needs of patients; (6) “lost time” to manage patients waiting for specialist access; (7) additional work strategizing ways to access specialist care for patients; (8) provider experience of burnout, frustration, and stress due to additional burden of delayed specialist care; and (9) recommendations for managing and accessing specialist care (Table 2).

**Table 2.** The nine emerging themes from the PCP survey responses.

Theme	Frequency of responses n (%)	Illustrative quote
Pervasiveness of problematic specialist wait times	27 (17%)	I’ve found that you have to beg to be considered to be seen.
Managing beyond scope while waiting for specialist care	35 (22%)	I now have to go outside my comfort zone and treat what I normally don’t feel is in the scope of my practice.
Consequences for patients due to specialist wait times	17 (11%)	Patients become sicker and in more pain and reduced mobility while they are waiting.
Managing patient expectations while waiting for specialist care	10 (6%)	Spent time explaining to a patient why a referral has not resulted in visits.
Scheduling repeat visits to meet needs of patients	14 (9%)	Repeat visits for symptoms management limits the number of new patients I can see.
“Lost time” to manage patients waiting for specialist access	25 (16%)	Additional time chasing appointments, redirecting referrals to other providers/jurisdictions (with time/effort to assess relative wait times/availability).
Additional work strategizing ways to access specialist care for patients	13 (8%)	I do not refer anyone to GI as it gives patients false hope that they ever would be seen. Rather utilize surgeons for scope.
Provider experience of burnout, frustration and stress due to additional burden of delayed specialist care	13 (8%)	Feel left out in the cold with very little support for me to support patients.
Recommendations for managing and accessing specialist care	2 (1%)	Need an e-consultant solution.

The pervasiveness of problematic specialist wait times was mentioned 27 times (17% of responses), with 93% of these responses indicating wait times as problematic for their practice, and only 7% indicating wait times having no effect on their practice.

The most common response from PCPs was long wait times for specialists resulted in greater management of patients themselves, often providing care outside their scope of practice: *“Greatly, I now have to go outside my comfort zone and treat what I normally don’t feel is in the scope of my practice.”* Another respondent expressed, *“I am required to provide advice/services beyond my level of expertise.”* This concern of working outside PCP scope of practice was mentioned 35 times (22% of responses).

PCPs raised concerns about patient suffering due to long wait times for specialists 17 times (11% of responses): *“Patients become sicker and in more pain and reduced mobility while waiting.” “Prepar[ing] patients for long waits”* for specialist care was mentioned 10 times (6% of responses).

When managing patients waiting for specialist care, PCPs mentioned scheduling more repeat visits for these patients (e.g. for monitoring and pain management), limiting opportunities for other and new patients: *“repeat visits for symptom management limits the number of new patients I can see.”* This concern of scheduling repeat visits was mentioned 14 times (9% of responses). PCPs also mentioned “*do[ing] a lot more administrative work to arrange follow-up etc.”* due to long wait times 25 times (16% of responses). Strategizing ways to access specialists was mentioned 13 times (8% of responses). Provider burnout, frustration and stress due to long patient wait-times for specialists was reported 13 times (8% of responses), saying it “*make[s] me feel more burnt out as I cannot appropriately care for patients.”* Finally, two physicians (1% of responses) reported recommendations for managing and accessing specialist care, with one respondent stating they *“need an e-consultant solution.”*

Reported challenges with wait times for specialist care varied by speciality. Twenty-eight PCPs reported various types of specialists with notably long wait times. Forty-seven references were made, mentioning long wait times for specialists including: psychology and psychiatry, gastroenterology, orthopaedics, internal medicine, rheumatology, haematology, surgery, endocrinology, neurosurgery, and urology (Table 3).

**Table 3.** Specialist types mentioned by responding PCPs as having long wait times.

Specialist type	Number of times mentioned n (%)
Psychology and psychiatry	17 (36.2)
Gastroenterology/Colonoscopy	12 (25.5)
Orthopaedics	5 (10.6)
Internal medicine	3 (6.4)
Rheumatology	3 (6.4)
Haematology	2 (4.3)
Surgery	2 (4.3)
Endocrinology	1 (2.1)
Neurosurgery	1 (2.1)
Urology	1 (2.1)

### Provider recommendations

Respondents provided five recommendations to manage or improve wait times for specialist care: (1) changing referral processes, (2) increasing follow-up and providing more care, (3) the need for extra time to be remunerated, (4) the need for e-solutions, and (5) the need for more providers.

***Changing referral processes***

The most discussed solution to enabling patient access to specialists was for PCPs to make changes to their usual referral processes. PCPs discussed referring patients to other locations with shorter wait times if patients are able to travel there: “*I send people to areas where wait times are not as long.”* PCPs also reportedly referred patients to the private system or to different specialists. For example, PCPs discussed referring patients to surgery rather than gastroenterologists because patients could get into surgery sooner. PCPs also discussed sending referrals in anticipation of patient needs and sending referrals to multiple locations: *“… sometimes sending duplicate referrals if [one specialist is] too long then send elsewhere …”*

PCPs also discussed challenges requiring support by specialists such as the lack of referral acknowledgement by many specialists. As one respondent noted, *“…my staff spend [more and more] time following up [on] referrals because patient hasn't heard anything. Cannot count the number of calls that this requires, all because we don't get … acknowledgement of a referral from many specialists …”* Respondents also shared the need for specialists to refer the patient to the next available specialist: *“… specialist[s] actually REFUSE … referrals and make us redirect … [should] be their job to send off to next available [specialist].”*

***Increasing PCP follow-up and providing more interim patient care***

PCPs discussed increasing the number of appointments with their patients as one strategy for mitigating harm caused by long specialist wait times: *“increased follow-up at times to monitor patient status while waiting for consult”* and offering more care to patients: “*need to see [patients] more frequently to follow issues that should be followed by specialists.”*

***Need for appropriate remuneration related to delayed specialist access***

Family physician respondents noted they were often working *“above [their] pay grade”* to provide care to patients waiting for specialist referral, and often spent more time researching diagnoses, management, and treatment options, saying they *“needed more research time to plan care.”* Physicians suggested extra time should be accordingly remunerated: *“Patients come in crisis, can take [a long time] with counselling which I'm poorly remunerated for!”*

***Need for e-solution***

One PCP expressed desire for e-solutions to facilitate specialist referrals. When asked how wait times for specialists affect the way you practice, one replied: *“horribly, need an e-consultant solution.”*

***Need for more providers***

Finally, one PCP suggested the wait times to see specialists is not due to specialists not working enough, suggesting more providers may be needed: *“wait times are excessive, not [due] to specialists, they are working [more and more]..*

## Interpretation

### Key findings

The aim of this study was to explore the pervasiveness of long wait times for specialists and the impact on PCPs in Nova Scotia. Findings from this research highlight the pervasiveness of these wait times, as majority of PCPs indicated wait times were problematic and negatively impacted both their patients and their practice. The most common concern mentioned by PCPs was long wait times resulting in management of patients with cares outside their scope of practice (e.g. mental health, internal medicine, and chronic disease care). PCPs expressed concern that patient wellness, pain, and mobility were worsening over time due to poor access to appropriate, good quality care. Additionally, PCPs reported having to manage patients’ expectations, preparing them for long wait times and explaining why the long wait, which often leads to managing patient frustration, anger, and stress. With long wait times, PCPs resorted to scheduling repeat appointments with these patients to monitor, manage pain and the emotional toll of waiting. PCPs disclosed these repeat visits take away from other patient access and a reduction in their ability to take on new patients. PCPs also mentioned experiencing increased workload (e.g. phone calls, administrative work, and research), resulting in lost time due to long wait times. Additionally, PCPs reported spending time strategizing and finding solutions to ensure their patients can access specialist care.

Participants identified short-term solutions or workarounds to these challenges including referrals to more accessible specialists, sending out multiple referrals, providing more care to patients in lieu of specialist care, and appropriate remuneration for this additional care. However, these solutions do not address the systemic challenges that need to be overcome for patients to have timely access to needed specialist care, and to unburden overwhelmed PCPs.

Participants identified several needed supports such as improved referral processes including better patient acknowledgement, specialist follow-up, and referral by the specialist to the next available specialist. Participants also identified needs for an e-solution to enable communication with specialists to better manage patient care while patients await in person appointments. There were also recommendations to increase the number of available specialists to address the backlog of wait times for care.

### Implications

The impact of long specialist wait times has implications across the Quintuple Aim.^20^

#### Provider experience

With increased burdens of managing patients outside their scope of practice, PCPs reported trying to manage patient suffering due to specialist wait times with repeat visits, managing patient expectations, and taking on extensive administrative tasks. These challenges lead to experiences of burnout, frustration, and stress. These negative outcomes of delayed access to specialists are concerning, as burnout among PCPs is associated with adverse outcomes including reduced provider quality of life, poor morale, increased physical and mental health concerns, increased medical errors,^24,26,27^ and new PCPs moving away from comprehensive family practice.^24^ Furthermore, PCP burnout is associated with adverse patient outcomes, as patients can experience lower quality of life, reduced patient satisfaction, reduced adherence to treatment, and the negative consequences resulting from physician errors.^27,28^

#### Patient experience

In addition to patient impacts such as increased frustration and stress due to high specialist wait-times,^29^ patients are found to experience negative outcomes due to PCP burnout such as lower quality of life, reduced patient satisfaction, reduced adherence to treatment, and the negative consequences from physician errors.^27,28^ Additionally, overburdening PCPs is associated with reductions in practice hours and increasing retirement,^30^ which have culminated in lost access to primary care itself relying on overcrowded walk-in clinics and emergency departments or paying for out-of-pocket private healthcare access.

#### Population health

As identified in this study, lack of timely access to specialists contributes to more work for PCPs, who may experience additional patient encounters with those waiting to see a specialist. This likely contributes to reduced ability to accept new patients into their practice or to meet the needs of other patients enrolled in their practice. This has implications for patient access to primary care, which is an ongoing challenge in the province of Nova Scotia.^31,32^

#### Value for money

Lack of timely access to specialists is associated with higher use of the primary care system, emergency departments, and walk-in clinics. Additionally, patients who are waiting to see a specialist may experience worsening health.^6,29^ Both consequences have implications for increased costs to the healthcare system.^33,34^ A recent study in Canada found although investments have been made in strategies to reduce wait times, Canadians continue to face high wait times.^29^ This suggests investments may need to be directed differently.

#### Health equity

Timely access to specialist care is important for patients who have co-morbidities. Often, equity-denied populations have unmet health needs, including those with disabilities, newcomers to Canada, and, in Nova Scotia, Indigenous and African Nova Scotian communities.^35,36^ Improving mechanisms for timely specialist referrals could help better support these populations.

### Future directions

Strategies and innovations for facilitating access to specialist care for patients are urgently needed, particularly during the COVID-19 pandemic recovery period. During the pandemic, non-essential specialist services were often suspended, PCPs were redeployed, and patients experienced barriers to accessing specialist care and often care was foregone^37,38^. As time goes on and patients experience worsened health outcomes, there has been a spike in demand for specialist care which may place additional burden on specialists and increase wait times. It has become evident that wait times for specialist services have become even longer since the pandemic. More research is needed to identify challenges posed by the pandemic, and implications across the Quintuple Aim.^20^ Fortunately, several innovations were introduced during the pandemic to support patient access to primary care^39^ and to facilitate consultations between PCPs and specialists.^40^ These initiatives should be evaluated to ascertain their usefulness across the Quintuple Aim.

### Strengths and limitations

Our population included both family physicians and nurse practitioners, with a response rate of 60% which is high compared to other studies involving healthcare providers.^23,24^ This research highlights the pervasiveness of long patient wait times for specialists within Nova Scotia, as well as the negative impacts on PCP practice. Future research could include data from other provinces and countries, to better understand how our healthcare structures cope, particularly at the interface between primary, secondary, tertiary, and quaternary care.

## Conclusion

The purpose of this study was to explore the pervasiveness and consequences of specialist wait times for patients and PCPs in Nova Scotia, Canada, and identify recommendations for improvement. PCPs identified pervasive negative impacts for themselves and their patients due to long wait times for specialist care in Nova Scotia. Although providers take numerous steps to manage their patients in the interim, system-level changes are needed to reduce the problems these wait times cause for patient and provider outcomes, population health, costs, and health equity. These changes are urgently needed, as these challenges likely were exacerbated during the COVID-19 pandemic with reduced access to care across the system. Fixing primary care, which is currently in crisis, will need to also address improvements in other levels of care for patients.
